# Intermittent fasting alleviates irradiation-induced neuronal mitochondrial damage in mice

**DOI:** 10.3389/fnut.2026.1815146

**Published:** 2026-05-14

**Authors:** Yaqi Li, Xingwen Fan, Yulei Pei, Kailiang Wu

**Affiliations:** 1 Department of Radiation Oncology, Shanghai Proton and Heavy Ion Center, Shanghai, China; 2 Shanghai Key Laboratory of Radiation Oncology, Shanghai, China; 3 Shanghai Engineering Research Center of Proton and Heavy Ion Radiation Therapy, Shanghai, China; 4 Department of Radiation Oncology, Fudan University Shanghai Cancer Center, Shanghai, China; 5 Department of Oncology, Shanghai Medical College, Fudan University, Shanghai, China; 6 Shanghai Clinical Research Center for Radiation Oncology, Fudan University Shanghai Cancer Center, Shanghai, China; 7 Department of Radiation Oncology, Shanghai Proton and Heavy Ion Center, Fudan University Cancer Hospital, Shanghai, China

**Keywords:** cognitive impairment, intermittent fasting, mice, mitochondria, radiation-induced brain injury

## Abstract

**Purposes:**

Radiation-induced brain injury (RIBI) is a common side effect of cranial radiotherapy. Intermittent fasting (IF) decreases the risk of Brain aging disease by affecting mitochondrial function. We examined the effects of IF on mitochondrial function, neurogenesis, morphology of hippocampal neurons and cognitive abilities in a whole-brain radiotherapy (WBRT) mouse model.

**Methods:**

6–8 weeks old male C57BL/6J mice received 10 Gy WBRT. IF group mice were subjected to cycles of 24 h food restriction followed by 48 h *ad libitum* access to food. Ultrastructural changes in the mitochondria of mice hippocampus and the ATP concentrations were evaluated at 6 h and 6 weeks post-irradiation. The hippocampal neurons were analyzed by Golgi staining with ImageJ. Animal behavior and newborn neurons (DCX+) were assessed 6 weeks after WBRT.

**Results:**

The proportion of swollen mitochondria in hippocampal neurons was significantly decreased in the IF+WBRT group than the WBRT (*p* = 0.0005) group at 6 h post-irradiation; At 6 weeks post-irradiation, the proportion of swollen mitochondria in the WBRT group remained significantly greater than that in the control group (*p* = 0.0003). ATP concentrations were higher in the IF+WBRT group than in the WBRT group (*p* = 0.0576). DCX+ numbers (*p* = 0.0049), neuron length (*p* = 0.0021), numbers of branches (*p* = 0.0262) and branch points (*p* = 0.0098) were significantly greater in the IF+WBRT group than in the WBRT group. Compared with the WBRT group, the IF + WBRT group presented significantly shorter open-arm time in the elevated plus-maze test (*p* = 0.0082).

**Conclusions:**

IF mitigates RIBI by reducing mitochondrial damage, promoting neurogenesis, and partially restoring ATP levels at the early stage, although its behavioral effects remain limited. The beneficial effect of IF is time-dependent and more prominent in the acute phase of RIBI.

## Introduction

Radiation-induced brain injury (RIBI) is a common side effect of cranial radiation therapy. Both early-stage and late-stage effects of such therapy can cause brain damage

such as memory loss, cognitive dysfunction, and even death. However, the mechanism through which RIBI develops after cranial radiotherapy is currently unclear, and there is no effective treatment.

The risk of many neurological diseases, including Alzheimer's disease (AD) (1), Parkinson's disease (PD) (2), stroke, and inflammatory diseases (3, 4), is significantly correlated with the presence of neuronal mitochondrial lesions. Mitochondrial dysfunction also plays an important role in the pathogenesis of AD (5). After whole-body X-ray irradiation, the number of copies of mitochondrial DNA (mtDNA) increases, but the expression of genes involved in ATP synthesis and mitochondrial dynamics decreases (6). Radiation may cause metabolic and dynamic changes in mitochondria in hippocampal tissue.

Intermittent fasting (IF) has been shown to increase brain function during brain aging and in individuals with neurodegenerative disease; it improves cognitive function (7) by affecting energy metabolism, oxidative damage, biogenesis, and dynamics in mitochondria (8). According to Longo and Mattson's research, IF alters brain neurochemistry and neuronal network activity in a way that optimizes brain function and peripheral energy metabolism 8. During fasting, a decrease in the systemic metabolism is associated with a decrease in the total number of liver mitochondria, leading to an increase in the mitochondrial respiratory control rate and ATP production, thereby supporting neuronal activity and inhibiting nerve damage (9). Increasing evidence suggests that in healthy adults, most systemic changes caused by IF, caloric restriction (CR), or food restriction (FR), such as reduced inflammation and improved glucose and fat metabolism, can prevent brain damage (10). Currently, there is a lack of research on the impact of IF on neuronal mitochondria after RIBI.

In this study, we treated whole brain radiotherapy (WBRT) mice with IF. We found that IF can partially alleviate mitochondrial damage in neurons, increase neuronal branching, and alleviate radiation-induced brain damage, especially in early stage.

## Methods and materials

### Mouse model and irradiation

6–8 weeks old Male C57BL/6J mice were purchased from GemPharmatech Co., Ltd. and housed in the Laboratory Animal Center of Fudan University Shanghai Cancer Center. The Ethics Committee of the Animal Center at Fudan University Shanghai Cancer Center approved the animal study protocol. The mice were housed under specific pathogen-free (SPF) conditions with a 12 h light/12 h dark cycle at 21–24 °C and 40%−60% humidity and were fed a standard chow diet at Fudan University Shanghai Cancer Center Laboratory Animal Center. Experimenters randomly allocated the mice into four groups: control, WBRT, IF, and IF+WBRT. To ensure data reliability, each experimental group contained 4–10 mice in all experiments. The IF followed a “1+2” pattern: they were fasted for 1 day (24 h) and fed a normal diet for 2 days (48 h) (11); during this time, they were given *ad libitum* access to water.

The mice were anesthetized via intraperitoneal injection of avertin and irradiated using a 6-MV X-ray linear accelerator (Primus Linear Accelerator, Siemens Healthcare, Forchheim, Germany). The source skin distance was 100 cm, and the dose rate was 2.5 Gy/min. The multi-leaf collimators were adjusted to open a treatment field of 1.5 cm × 40 cm for five mice that underwent WBRT simultaneously. The mice were then subjected to WBRT at a dose of 10 Gy in a single fraction as previously reported in the literature (12, 13), and experiments were performed at 6 h and at 6 weeks after irradiation.

### Transmission electron microscopy

The mice were anesthetized and perfused with 4% paraformaldehyde and 2%−3% glutaraldehyde fixative. The hippocampal region was dissected and cut into 1-mm3 pieces; the pieces were fixed at 4 °C for 4 h and washed with 0.1 M phosphate buffer. Post-fixation processing with 1% osmium tetroxide was performed for 2–3 h, followed by washing with buffer. The tissue was dehydrated through an ethanol and acetone series, infiltrated with acetone-resin mixtures, and embedded in epon resin. The resin was cured at 37 °C, 45 °C, and 60 °C. Ultrathin sections (70 nm) were cut by Leica UC7, stained with uranyl acetate and lead citrate, and examined via TEM (JEM-1230, 80 Kv, JEOL Ltd.). Three to six neurons were randomly selected from each sample, and images were captured at 5,000 × , 20,000 × , and 50,000 × for mitochondrial analysis.

### Analysis of mitochondrial morphology

The extent of mitochondrial damage in the mouse hippocampus was assessed and graded using electron microscopy to evaluate the degree of morphological alteration in the mitochondria. Mitochondrial morphology was examined on the basis of the space between outer and inner mitochondrial membrane, known as the intermembrane space, and the mitochondrial density, which refers to the number of mitochondria per unit area within the hippocampal tissue. The mitochondria were classified as altered according to criteria that have been validated in previous morphological studies (14), as follows: (0) normal; (1) normal-vesicular: significantly decreased electron density of the matrix (dilution, vacuolization, cavitation); (2) vesicular: fragmented and ballooned cristae (intracristal swelling); (3) vesicular-swollen: partial or complete separation of the outer and inner membranes; and (4) swollen: mitochondrial swelling. Mitochondrial morphology was systematically evaluated at each level according to these criteria.

### DCX (Doublecortin) immunohistochemistry

After 6 weeks of treatment, DCX-positive (DCX+) cells in the mice were measured to assess neurogenesis. As previously described, 40-μm-thick coronal sections encompassing the hippocampus (covering an area from 3.2 to 4.0 mm posterior to bregma) were prepared. The tissue sections were initially deparaffinized and rehydrated. Antigen retrieval was performed by incubating the sections in sodium citrate buffer (10 mM sodium citrate, 0.05% Tween-20, pH 6.0) to expose the epitopes. To minimize nonspecific binding, the sections were incubated in a blocking solution (BSA). Primary antibodies specific to the target antigen were then applied and allowed to bind. The sections were incubated with a mouse antibody against DCX (1:200; Santa Cruz Biotechnology Inc., Dallas, TX, USA) at 4 °C overnight. The sections were then incubated with a secondary antibody (1:200, biotinylated goat anti-mouse; Jackson Immuno Research Laboratories Inc., West Grove, PA, USA) for 1 h at room temperature, counterstained (Streptavidin-conjugated horseradish peroxidase binds specifically to biotin and DAB substrate), dehydrated, and mounted for microscopic analysis. Images were captured using an E600FN Neurolucida system (Nikon Corp., Tokyo, Japan) equipped with a 20X objective. Image J software (Fiji, National Institutes of Health, Bethesda, MD, USA) was used to calculate the number of DCX+ cells and the length of the dentate gyrus. The density of DCX+ cells was then calculated as the number of positive cells per unit length of the dentate gyrus.

### Golgi staining for determining neuronal length and dendritic spine density

At 6 weeks posttreatment, the mice were anesthetized with 2.5% avertin (five animals per group), and their brain tissue was removed for Golgi staining. The brain tissue samples were fixed in potassium dichromate, followed by immersion in silver nitrate to impregnate the tissue and highlight the neurons. After being rinsed, dehydrated, and cleared in xylene, the samples were mounted in resin for microscopic examination. Five to eight neuronal cells were randomly selected from each sample for analysis, and approximately 10 segments of dendritic spines were extracted for dendritic spine analysis. Spine density was calculated as spine count divided by dendritic length. For analysis, high-resolution images of Golgi-stained neurons were acquired and imported into ImageJ with plugins (Simple Neurite Tracer). Neurons were traced by Simple Neurite Tracer to measure total neuronal length as previously reported (15). For Dendritic spines, preprocessing enhanced contrast and reduced noise by Adobe Photoshop 2024 (Version 25.4, Adobe Inc., San Jose, CA, USA) and ImageJ. Images were adjusted to grayscale and subjected to curve adjustment using Adobe Photoshop 2024 (Adobe Inc., San Jose, CA, USA). Subsequently, the processed images were imported into ImageJ software, where they were converted from RGB to grayscale and further subjected to binarization. Dendritic spines were detected by thresholding and use of the ‘Analyze Particles' function, with spine density calculated by dividing the spine count by the dendritic length (16). The data were exported for further statistical analysis.

### ATP concentration

The mice were anesthetized with 2.5% avertin at 6 h, 1 week and 6 weeks posttreatment. The hippocampus was isolated for analysis of the ATP concentration. An appropriate volume of double-distilled water and protease inhibitors was added on the basis of the hippocampal tissue weight, followed by homogenization of the tissue. The samples were centrifuged at 3,500 rpm for 10 min, and the supernatant was collected for testing. Following the instructions of the ATP reagent kit (Nanjing Jiancheng Bioengineering Institute, A095-1-1), by using the phosphomolybdic acid colorimetric method to detect the amount of phosphorus generated and calculate the quantity of ATP content, control tubes, standard tubes, blank tubes and test tubes were set up. We prepared samples, induced reactions (substrate solution, promotor, precipitation agent, chromogen), and terminated the reactions. Finally, the samples were incubated at room temperature for 5 min, and the absorbance at 636 nm of the contents of each tube was measured using a light path of 0.5 cm. The results were used to calculate the ATP concentration.

### Behavioral tests

^*^All behavioral tests were performed at 6 weeks post-irradiation, and the mice were treated by numbers. Schematic diagrams of each behavioral test are shown in Supplementary Figure 1.

#### Morris water maze (MWM) experiment

MWM assays are routinely performed to assess the effects of IF on WBRT. The water maze was 150 cm in diameter and approximately 60 cm in height and was filled with water to a depth of approximately 40 cm. The water temperature was maintained at approximately 22–25 °C. A platform was placed in the pool below the water, and an overhead camera was mounted above the pool and used to track the movements of the mice. Each mouse underwent four trials for 7 days, allowing 60 s for each trial. On the 8^th^ day, the mice were allowed to swim freely in the pool for 60 s. The time spent in the target quadrant and the number of times the platform location was crossed were recorded (Med Associates). The records were used for further analysis.

#### Novel object recognition test

The novel object recognition test was conducted as previously described (12). The test had three phases: an adaptive phase, a familiarization phase, and a test phase. In the adaptive phase, the mouse was placed in an empty box and allowed to move freely for 5 min. The mice were then returned to their original cage, and the empty boxes were cleaned with 70% alcohol. In the familiarization phase, two identical objects were placed in the box, each at a distance of 5 cm from the box wall. The same objects were randomized in advance for exposure to different groups of mice. In the test phase (after 24 h), one of the objects was replaced with an object of a different shape. The novel object was positioned randomly in the box. A stopwatch was used to measure the amount of time the mouse spent exploring each of the two objects. If the total exploration time reached 20 s or if the experimental time reached 5 min, the experiment was stopped, and the amount of time spent was recorded. The preference ratio (time spent exploring the novel position/total exploration time) after 1 min, 3 min, and 5 min was calculated.

#### Fear conditioning test

The fear conditioning test was used to evaluate fear learning and memory in mice after 6 weeks of WBRT. A MED-APA-D1R apparatus manufactured by Med Associates Inc. (Fairfax, VA, USA) was used. The test comprises three phases: a training phase to establish associative fear memory, a context testing phase to assess hippocampus-dependent contextual fear memory, and a cue testing phase to evaluate amygdala-dependent cued fear memory. During training, after explore the conditioning chamber for 3 min, each mouse was exposed to auditory, visual, and electrical stimuli (1–2 s foot shocks at 0.2–0.5 mA). During the context test, the mice were placed in the chamber without stimulation for 5 min to permit assessment of context-associated fear conditioning. During the cue test, mice were initially placed in the chamber without stimulation for 3 min; then, 3 min of auditory stimulation was provided to permit evaluation of amygdala-associated fear conditioning.

#### Elevated plus maze (EPM)

To reduce stress, the animals were acclimated to the behavioral laboratory environment for at least 3 h prior to the experiment. The mice were gently placed in the central area of the maze, facing an open arm and with their backs toward the experimenter, and SuperMaze animal behavior analysis software was used to track animal movement within the EPM for a period of 5 min and to automatically calculate the relevant metrics: the latency to the first open-arm entrance, the number of open-arm entries (OE), and the time spent in the open arms (OT). After the experiment, the mice were returned to their home cages and marked. The maze was cleaned with alcohol and paper towels prior to the next trial.

#### Open-field test

In this study, the open-field test was performed to assess the effects of IF on locomotor activity and anxiety-like behavior in mice receiving WBRT. The open-field apparatus used was a 50 x 50 cm square arena with 30-cm high black walls (MED-VOF-RS; Med Associates), as previously described (12). The animals were transported to the testing room and left undisturbed for 30 min before the test. Each mouse was placed in the middle of a peripheral zone of the arena facing the wall and allowed to explore the apparatus freely, with the experimenter out of the mouse's sight. A video tracking system was used to record the movement of the mice. The videos were analyzed via Activity Monitor (Med Associates Inc.). The entire exploration phase lasted for 5 min. The number of times the mice entered the central area, the distance they moved, and the duration of their stay in the open field were recorded.

### Statistical analysis

The data are expressed as the means ± standard errors of the means (SEMs). To minimize experimental error, statistical outliers were excluded from the analysis. Statistical analyses were performed using the unpaired Student's *t*-test for comparisons between two groups. For comparisons involving multiple groups, one-way ANOVA with repeated measures was applied, followed by Bartlett's test where appropriate. All the statistical procedures were conducted using GraphPad Prism 9.0 software (GraphPad Software Ltd.). *p*-values less than 0.05 were considered to indicate statistical significance.

## Results

**Figure 1:**
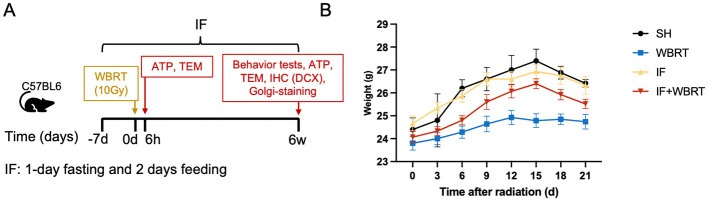
Mice modeling, experiments, and weight changes (*n* = 10 per group). **(A)** Timeline illustration of the experimental design: mice were sacrificed after 6 h and 6 weeks. IF was conducted 1-day fasting+2 days casual diet. **(B)** Dynamic change of the mice weight in each 3 days after radiation, and remained stable after day 21. *P*-values were considered statistically significant. SH, sham control; WBRT, whole brain radiotherapy; IF, intermittent fasting; IF+WBRT, intermittent fasting+ whole brain radiotherapy; TEM, transmission electron microscope; ATP, adenosine triphosphate; IHC, immunohistochemistry; DCX, Doublecortin.

To investigate the effect of IF on RIBI, we established a mouse model (Figure 1A). The mice used in the model were sacrificed 6 h or 6 weeks after WBRT. After WBRT, we monitored the weights of the mice. We observed significant differences in weight between the mice exposed to WBRT and those in the other groups beginning on day 9. The weights of the mice in the WBRT group treated with IF were greater than the weights of the mice in the WBRT group. The difference was most significant on the 15th day. There was no significant difference in weight between the mice in the IF group and those in the control group (Figure 1B).

**Figure 2:**
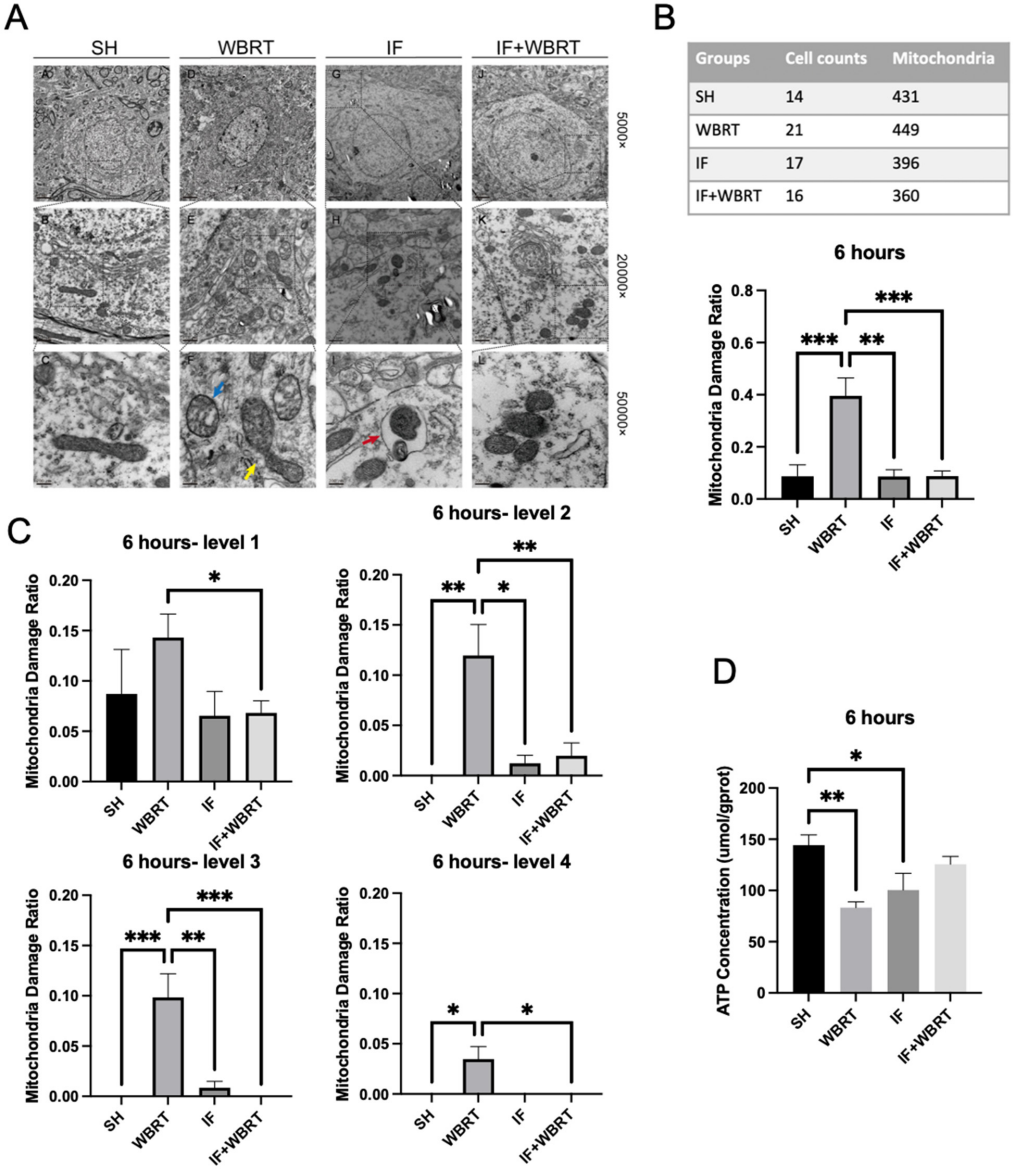
The morphology of mitochondria and ATP concentration after 6 h post-irradiation (*n* = 4). **(A)** Mitochondrial morphology in hippocampal neurons was analyzed using TEM at magnifications of 5,000 × , 20,000 × , and 50,000 × . **(B)** The fraction of damaged mitochondria in each group based on TEM images at all levels. **(C)** The fractions of mitochondria exhibiting different levels of mitochondrial damage at each level in 6 h post-irradiation. **(D)** The ATP concentration in the hippocampus in 6 h. All data were analyzed using one-way ANOVA. *P*-values were considered statistically significant, *P < 0.05, [**]*P* < 0.01, ***P < 0.001, ***P < 0.0001. TEM, transmission electron microscope; SH, sham control; WBRT, whole brain radiotherapy; IF, intermittent fasting; IF+WBRT, intermittent fasting+ whole brain radiotherapy.

We subsequently employed transmission electron microscopy to observe the mitochondria within the neurons. The neurons exhibited normal gross morphology; the nuclei were large and distinct, with clearly defined nuclear membranes. The cytoplasm contained abundant organelles, including rough endoplasmic reticulum, the Golgi apparatus, and mitochondria. The mitochondria were particularly numerous, and their morphology was analyzed in detail. In normal neurons, mitochondria typically display smooth, continuous outer membranes with densely and uniformly arranged cristae. In contrast, mitochondria within damaged neurons presented altered length–width ratios, often approaching 1:1, and significant swelling. In some of the samples, the double-membrane structure appeared markedly disrupted, with some membranes showing discontinuities or fragmentation. The mitochondrial cristae were also disrupted, and in severe cases, they appeared either vacuolated or granular. We found that the WBRT group, which received WBRT at a dose of 10 Gy for more than 6 h, exhibited a significant increase in fraction of damaged mitochondria (*p* = 0.0019). In contrast, the brains of the mice subjected to WBRT following IF intervention showed no difference in fraction of damaged mitochondria compared with those of the control group (*p* < 0.0001) and and there was a significant decrease in a fraction of damaged mitochondria compared with mice that underwent WBRT alone (*p* = 0.0005) (Figures 2A, B). We graded our samples on the basis of the grading criteria for mitochondrial morphological damage reported in the previous literature (14) (Supplementary Figure 2). There were significant differences between the WBRT group and the IF + WBRT group in fractions of mitochondria exhibiting different levels of mitochondrial damage (grade 1 injury, *p* = 0.0134; grade 2, *p* = 0.0078; grade 3, *p* = 0.0002; grade 4, *p* = 0.0171). IF reduced the degree of mitochondrial damage (Figure 2C). The ATP concentration in the tissues was also analyzed. 6 h after WBRT, the ATP content of the tissue was significantly decreased and was markedly lower than that in the control group (*p* = 0.0049). IF intervention reduced the decrease in ATP (*p* = 0.0576) but did not result in significant differences (Figure 2D).

**Figure 3:**
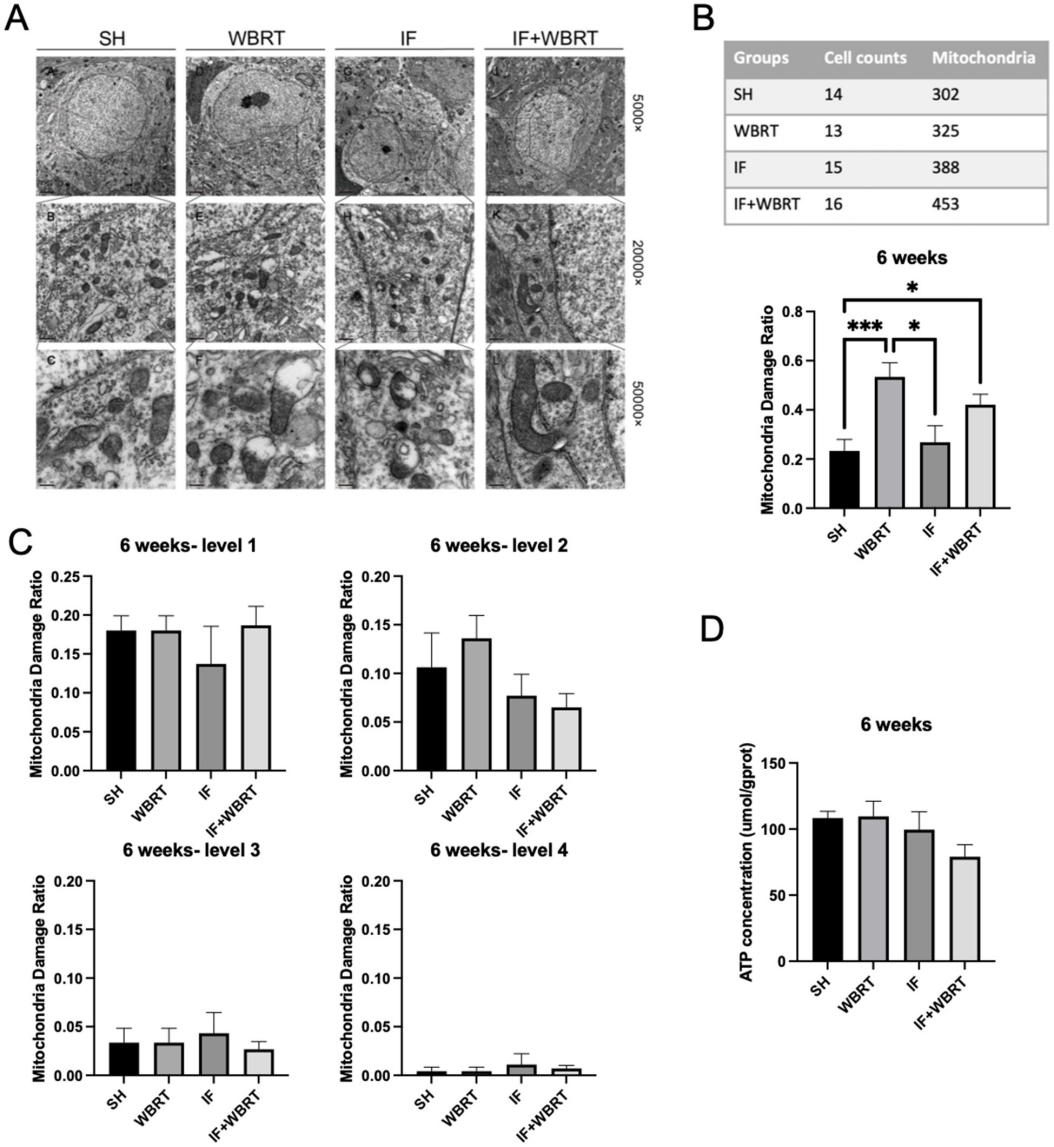
The morphology of mitochondria and ATP concentration at 6 weeks post-irradiation (*n* = 4 per group). **(A)** Mitochondrial morphology in hippocampal neurons was analyzed using TEM after 6 weeks. **(B)** The fraction of damaged mitochondria in sham control, WBRT, IF, and IF + WBRT based on TEM images. **(C)** The fractions of mitochondria exhibiting different levels of mitochondrial damage at 4 levels. **(D)** The ATP concentration in the hippocampus in 6 weeks. All data were analyzed using one-way ANOVA. *P*-values were considered statistically significant, *P < 0.05, ***P < 0.001, ****P < 0.0001. TEM, transmission electron microscope; SH, sham control; WBRT, whole brain radiotherapy; IF, intermittent fasting; IF+WBRT, intermittent fasting+ whole brain radiotherapy.

Representative TEM images illustrating the observed morphological damage to the mitochondria in hippocampal neurons at 6 weeks posttreatment are shown in Figure 3A. In mice that had received WBRT at 10 Gy 6 weeks previously, the degree of mitochondrial damage was significantly greater than that in the control group (*p* = 0.0003). In the IF + WBRT group, mitochondrial damage differed significantly from that in the control group (*p* = 0.0453) but not from that in the WBRT group (*p* = 0.3927) (Figure 3B). The fractions of mitochondria exhibiting different levels of mitochondrial damage at 6 weeks post-WBRT are presented in Figure 3C. Between the treatment groups, there were no statistical significant differences in the fractions of mitochondria exhibiting different levels of damage. (grade 1, *p* = 0.7719; grade 2, *p* = 0.2121; grade 3, *p* = 0.9200; grade 4, *p* = 0.8156). Notably, at 6 weeks after WBRT, no significant differences were observed in ATP content in brain tissue among the four groups (*p* = 0.1330) (Figure 3D).

**Figure 4:**
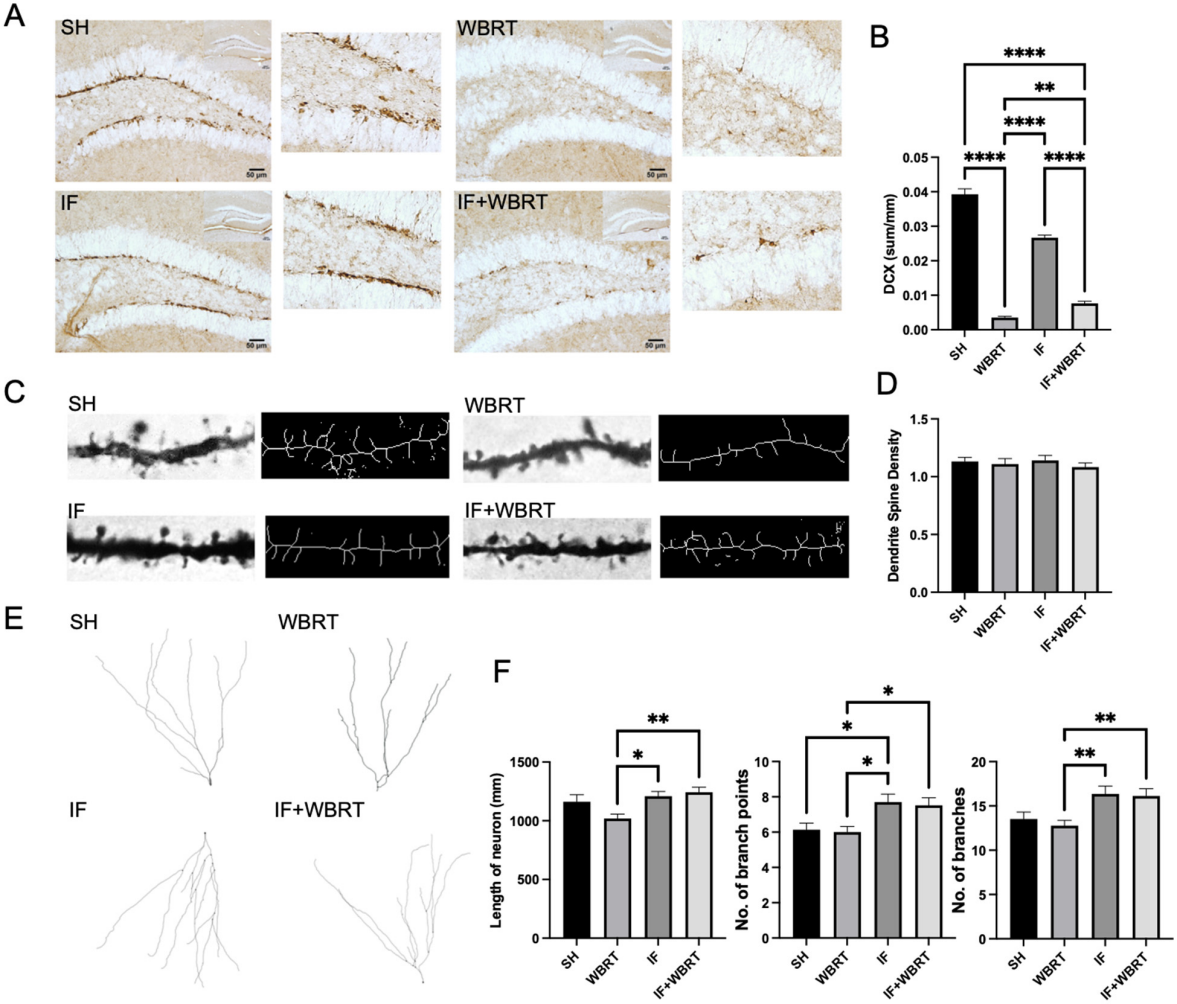
Fasting could increase the DCX+ and branches of the neurons after brain irradiation (*n* = 5 per group). **(A)** The IHC imaging of sham control, WBRT, IF, and IF + WBRT; **(B)** the quantification of DCX+ neurons in each group; the representative images of **(C)** neurons dendritic spine and **(E)** neurons skeleton by Image J; the quantitative analysis of Golgi-stained neurons in the dentate gyrus included measurements of **(D)** dendritic spine density, **(F)** length of neuron, branch points and number of branches. All data were analyzed using one-way ANOVA. *P*-values were considered statistically significant, *P < 0.05, **P < 0.01, ****P < 0.0001. SH, sham control; WBRT, whole brain radiotherapy; IF, intermittent fasting; IF+WBRT, intermittent fasting+ whole brain radiotherapy.

We then conducted a study of newborn neurons in the dentate gyrus of the hippocampus. Representative images of newborn neurons in the hippocampi of animals in the four experimental groups are shown in Figure 4A. Consistent with other studies, the number of newborn neurons in the dentate gyrus was significantly lower in animals that had received WBRT than that in the control group (*p* < 0.0001), whereas the number of newborn neurons in the IF intervention group was significantly greater than that in the control group (*p* < 0.0001). Compared with the WBRT group, the IF + WBRT group presented significantly more newborn neurons (*p* = 0.0049), indicating a trend toward recovery (Figure 4B).

We next used Golgi staining to examine the neurons in the dentate gyrus of the hippocampus and ImageJ software to statistically analyze the results. A comparison of the dendritic spine density in the dentate gyrus of the hippocampus in mice in the four experimental groups is shown in Figure 4C. There were no significant differences in the spine densities of hippocampal neurons in the four groups of mice (*p* = 0.7958) (Figure 4D). A comparison of the neuron skeleton in the dentate gyrus of the hippocampus in mice among the four groups is shown in Figure 4E. We found substantial differences in neuronal length, the number of dendritic branch points, and the number of dendritic branches (*p* = 0.0025, *p* = 0.0015, and *p* = 0.0025, respectively, using one-way ANOVA): the IF group had significantly more branch points than did the control group (*p* = 0.0435), and neuronal length, number of branch points, and number of branches were greater than those in the WBRT group (*p* = 0.0212, *p* = 0.0171, and *p* = 0.0092, respectively). The IF + WBRT group also showed significantly greater values for these parameters than did the WBRT group (*p* = 0.0021, *p* = 0.0262, and *p* = 0.0098, respectively) but no significant differences in these parameters from the control group (*p* = 0.5845, *p* = 0.0668, and *p* = 0.0833, respectively) (Figure 4F).

**Figure 5:**
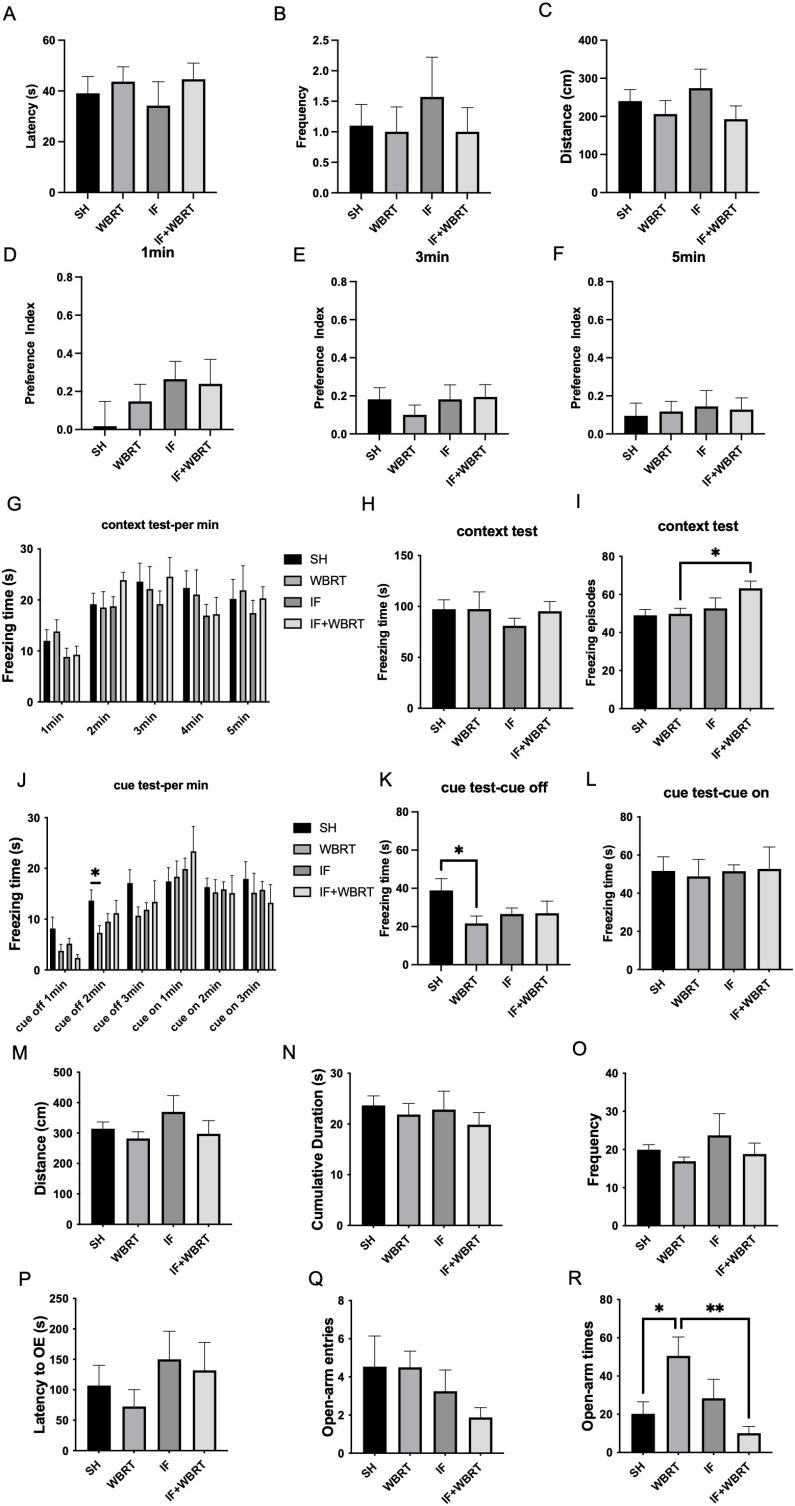
Mice after 6 weeks WBRT were used to conduct behavioral experiments (*n* = 10 per group). **(A, B, C)** Water maze; **(D, E, F)** New object recognition; **(G, H, I)** Context test data and **(J, K, L)** Cue data of the conditional fear experiment; **(M, N, O)** The distance they move, the number of times mice enter the central area, and the duration of their stay in the open field experiment; **(P, Q, R)** The latency to OE, numbers and times of mice entering the open arm in the elevated plus maze experiment. All data were analyzed using Student's *t*-test and one-way ANOVA. *P*-values were considered statistically significant, *P < 0.05, **P < 0.01. SH, sham control; WBRT, whole brain radiotherapy; IF, intermittent fasting; IF+WBRT, intermittent fasting+ whole brain radiotherapy.

Finally, we conducted behavioral experiments on the mice at 6 weeks posttreatment. In the water maze experiment, there were no significant differences in latency, number of platform crossings, or distance traveled in the target quadrant among the control, WBRT, IF, and IF + WBRT groups (*p* = 0.7347, *p* = 0.8034, *p* = 0.4464, respectively) (Figures 5A, B, C). In the novel object recognition test, we measured the animals' preference index for the new object at 1 min, 3 min, and 5 min; the results revealed no significant differences (*p* = 0.4154, *p* = 0.7252, *p* = 0.9605, respectively) (Figures 5D, E, F).

Figure 5G displays the freezing time of the mice per minute during the context test in the conditional fear experiment in which electric shock was employed. The average number of total freezing episodes for the animals in in the control, WBRT, IF, and IF + WBRT groups were 97.32 s, 97.38 s, 81.09 s, and 95.28 s, respectively (Figure 5H) (*p* = 0.1214), and the average total freezing episodes for the animals in those groups were 49, 49.8, 52.7, and 63.2, respectively (Figure 5I). And the freezing episodes of IF+WBRT were significantly increased than in WBRT group (*p* = 0.0132). The change in the freezing time of the mice per minute in the cue test is illustrated in Figure 5J. In the cue-off state, the freezing time of the mice in the WBRT group was significantly shorter than that of the control group (*p* = 0.0287), whereas there was no significant difference in freezing time between the IF + WBRT group and the WBRT group (*p* = 0.4854) (Figure 5K). In the cue-on state, there was no significant difference in freezing time among the four groups (*p* = 0.9893). The average freezing times within 3 min of cue-on were 51.7 s, 48.8 s, 51.5 s, and 52.7 s, respectively (Figure 5L).

In the open field test, there were no significant differences among the four groups in terms of the number of entries into the central area, the distance traveled, or the dwell time (*p* = 0.3374, *p* = 0.4144, and *p* = 0.7385, respectively) (Figures 5M, N, O). In the elevated plus maze test, there was no significant difference in latency to enter the open arms (*p* = 0.4957), although the mean values for the four groups were 106.8 s, 72.5 s, 150.0 s, and 131.8 s (Figure 5P). No differences were observed among the four groups in the number of open-arm entries (*p* = 0.4257) (Figure 5Q). However, a significant difference in the total time spent in the open arms of the maze was found (*p* = 0.0082) (Figure 5R). Compared with the mice in the control group, the mice that received WBRT spent significantly more time in the open arms of the maze (*p* = 0.0257), and the IF + WBRT group presented a significantly shorter dwell time than the WBRT alone group did (*p* = 0.0082).

## Discussion

Cranial radiotherapy is an essential treatment for head and neck malignancies as well as for metastatic brain lesions. However, it is often associated with a significant side effect, radiation-induced brain injury (RIBI). Notably, the onset of substantial side effects is typically delayed, emerging only after the optimal window for intervention has passed. The complex mechanisms underlying RIBI remain incompletely understood. Our study revealed that WBRT markedly suppresses hippocampal neurogenesis and induces measurable mitochondrial dysfunction in mature neurons, ultimately leading to memory deficits in murine models. Furthermore, our findings suggest that intermittent fasting may provide a potential degree of protection against the detrimental effects of WBRT, mainly during the early stage.

Previous studies have shown that neuronal mitochondria are crucial in the occurrence and development of brain-related diseases. Various irradiation model studies have confirmed that radiation significantly impacts the bioenergetics of cellular mitochondria (6, 17). In this study, ATP levels in the hippocampus decreased significantly within a short period (6 h) after WBRT, and mitochondria underwent significant morphological changes. In previous studies, IF has been reported to improve cognitive function and the structure of the brain (18), and it can increase mitochondrial dynamics, biogenesis, and metabolism (8). IF intervention in mice may protect mitochondria and reduce the effect of radiation on mitochondria in the early stage. However, in the middle-term (1 week), ATP levels in the hippocampi of the irradiated mice were still lower than those in the control group, and IF did not appear to promote ATP synthesis. In the late stage (6 weeks), the ATP content of each group returned to normal, and there were no significant differences between the groups. Our results demonstrate that IF has specific effects on the function of mitochondria at the early stage and morphology of mitochondria in mouse hippocampal neurons in the WBRT model.

Understanding the effects of IF on animals at both the cellular and individual cognitive levels is crucial. The literature contains reports that IF can alleviate neurodegeneration by increasing mitochondrial protein synthesis and ATP production, reducing apoptosis, and increasing neuronal survival rates (19, 20). Our findings confirm that IF increases the production of new neurons in the dentate gyrus of the hippocampus after WBRT. Moreover, it significantly increased the length and branching points of existing neurons in WBRT mice. These findings provide additional evidence supporting the hypothesis that IF serves as a means of alleviating RIBI. However, the density of dendric spine in the IF group mice did not change significantly; this contrasts with the findings of Ngo et al., who demonstrated that acute fasting could increase dendric spine density (21). Variations in radiation dose and fasting protocols may account for this discrepancy. In the present study, the number of DCX+ cells was significantly lower in the fasting group than in the control group; this is inconsistent with the findings reported by Dias et al. (22), whose results suggested that IF markedly increases the number of DCX+ cells. This discrepancy may be attributed to differences in the experimental protocols used. Dias et al. employed a regimen of 1 fasting day followed by 1 day of regular diet, whereas our study utilized a pattern of 1 fasting day followed by 2 days of regular diet. Despite these differences, our findings indicate that the applied fasting regimen partially mitigated the adverse effects that were otherwise induced by WBRT on neurogenesis and neuronal morphology, rather than exerting a full rescue effect.

In animal behavior experiments, mice have shown some cognitive benefits from IF in novel object recognition tasks and conditioned fear experiments. However, these effects are not observed consistently across all behavioral tests. Notably, in the elevated plus maze, mice in the IF+WBRT group spent less time in the open arms than those in the WBRT group, suggesting increased anxiety-like behavior rather than functional improvement. Similar trends were observed in the open-field test, which were consistent with previous findings (19, 20). These observations indicate that IF does not exert a clear, consistent protective effect on radiation-induced brain injury at the behavioral level. Some outcomes even suggest a potential adverse tendency in terms of anxiety-related behaviors. Therefore, the behavioral evidence supporting the neuroprotective effects of IF is limited and inconsistent. The variability in the observed outcomes may be related to the specific IF protocols used, including the duration of the fasting period and the overall fasting schedule. Additionally, the existing literature indicates that different types of IF can have distinct effects (21, 23). Therefore, further research is essential to determine the optimal IF regimen for protection against radiation-induced brain injury (RIBI).

In conclusion, we observed that IF exerts a potential protective effect on mitochondrial integrity in hippocampal neurons, as well as mitochondria function in WBRT model. Given its economic value, simplicity and ease of implementation, IF holds promise as a potential intervention for the amelioration of RIBI.

## Data Availability

The raw data supporting the conclusions of this article will be made available by the authors, without undue reservation.
