# Supplementary material for: Intermittent fasting alleviates irradiation-induced neuronal mitochondrial damage in mice
Source: Front Nutr. 2026 May 14;13:1815146. doi: 10.3389/fnut.2026.1815146 (PMC13216467; doi:10.3389/fnut.2026.1815146)
Supplement: Supplementary file 1 [file Supplementary_file_1.docx]

*
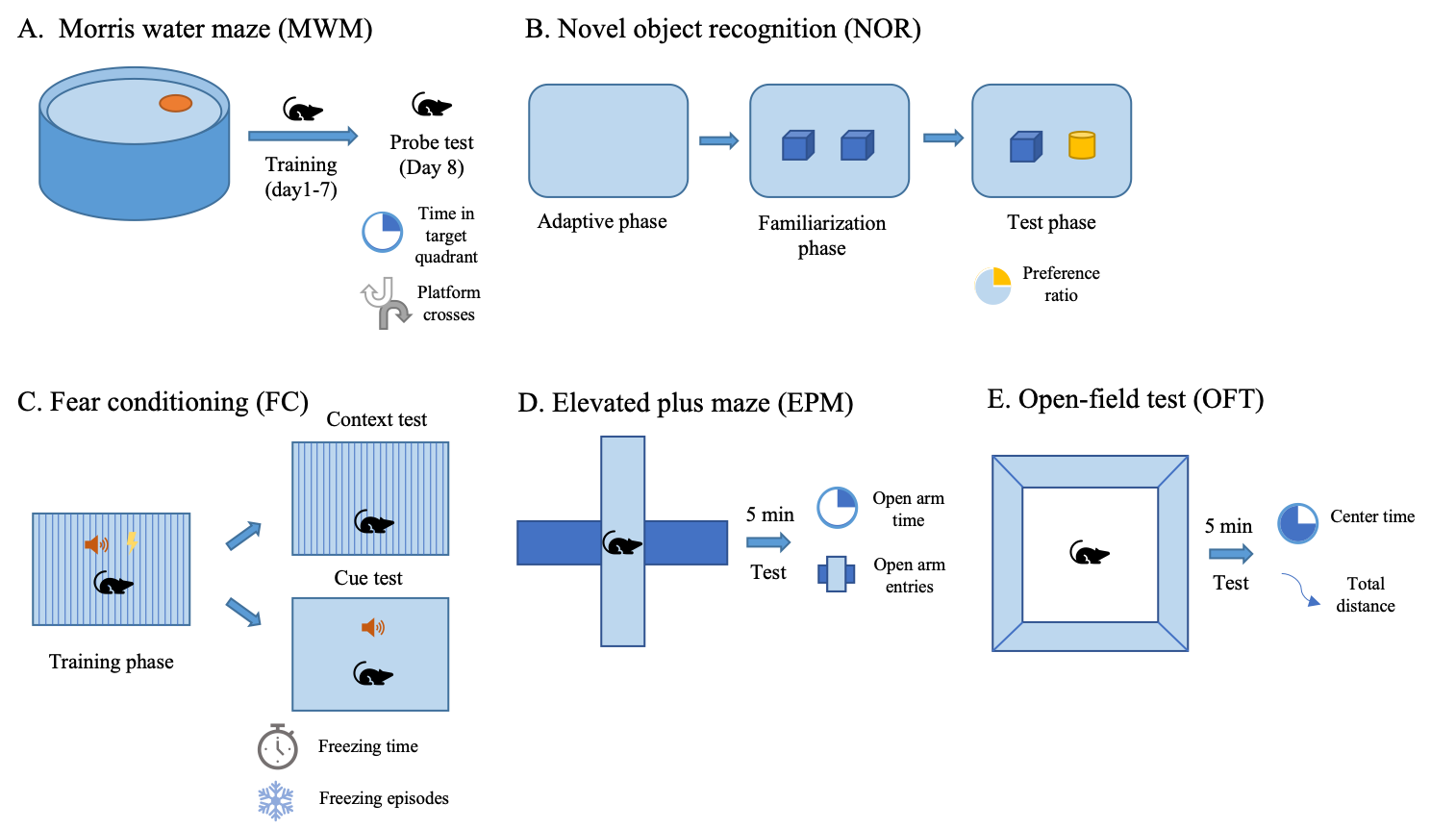
*

Supplementary figure 1. Schematic drawing for each behavioral experiment. (A). Morris water maze (MWM) experiment; (B). Novel object recognition test; (C). Fear conditioning test; (D). Elevated plus maze (EPM); (E). Open-field test.


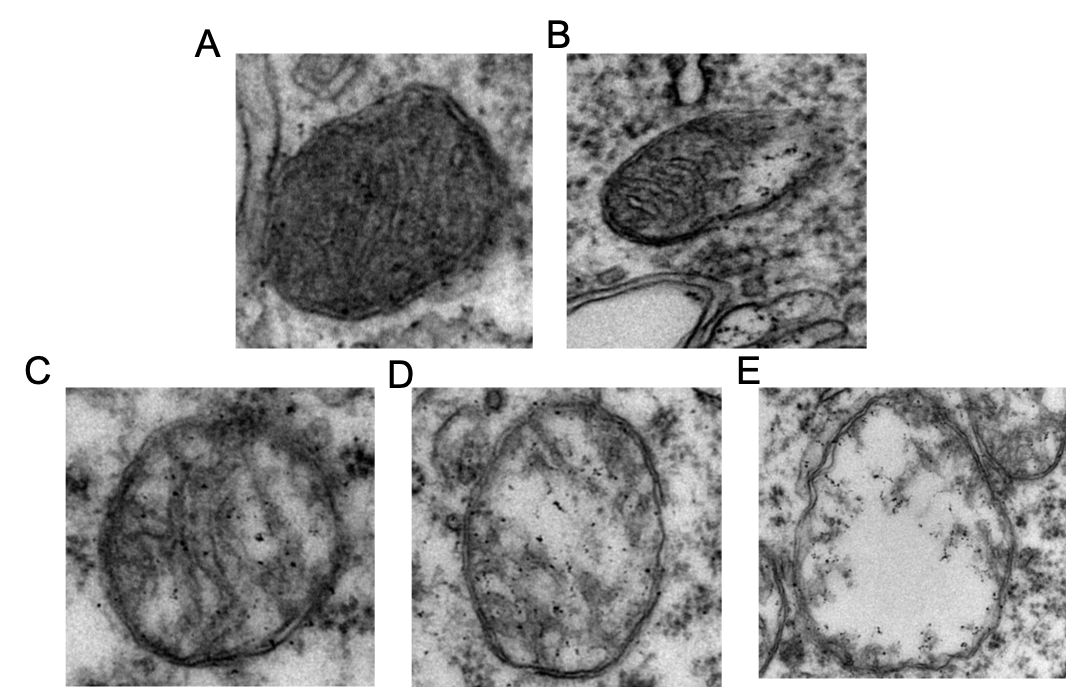


Supplementary figure 2. Representative diagram of mitochondrial damage at all 5 levels. (A) normal, grade 0; (B) normal-vesicular, grade 1 (C) vesicular, grade 3; (D) vesicular-swollen, grade 4; (E) swollen, grade 5.

Supplementary figure 3. The ATP content in the hippocampus one week after irradiation. SH, sham control; R, whole brain radiotherapy; F, intermittent fasting; F+R, intermittent fasting+ whole brain radiotherapy. *P<0.05, ***P<0.001.
